# Survival predictors of preterm neonates: Hospital based study in Iran (2010-2011)

**Published:** 2013-12

**Authors:** Ladan Haghighi, Marzieh Nojomi, Behnaz Mohabbatian, Zahra Najmi

**Affiliations:** 1 *Department of Obstetrics and Gynecology, Iran University of Medical Sciences, Tehran, Iran. *; 2 *Department of Community Medicine, School of Medicine Iran University of Medical Sciences, Tehran, Iran*

**Keywords:** *Preterm*, *Neonate*, *Survival*, *Mortality*, *Iran*

## Abstract

**Background:** Preterm birth (PTB) is responsible for 70% of neonatal mortalities. Various factors influence the risk of neonatal mortality in different populations.

**Objective: **Our objective was to evaluate neonatal survival rate of preterm infants, and to define its predictors in Iranian population.

**Materials and Methods:** This retrospective cohort study included all preterm (26-37 weeks) infants (n=1612) born alive in Shahid Akbar-abadi university hospital, during one year period (April 2010-2011). These infants were evaluated for fetal-neonatal, maternal, and pregnancy data. Survival analysis was performed and viability threshold and risk factors of neonatal mortality were evaluated.

**Results:** Total overall mortality rate was 9.1%. Survival rate were 11.11% for extremely low birth weights (LBW) and 45.12% for very early PTBs. The smallest surviving infant was a 750 gr female with gestational age (GA) of 30 weeks and the youngest infants was a 970 gram female with GA of 25weeks plus 2 days. History of previous dead neonate, need to cardio-pulmonary resuscitation (CPR), need to neonatal intensive care unit (NICU) admission, postnatal administration of surfactant, presence of anomalies, Apgar score <7, multiple pregnancy, non-cephalic presentation, early PTB, very early PTB, LBW, very low birth weight (VLBW) and extremely low birth weight (ELBW), were risk factors for mortality in preterm neonates.

**Conclusion:** Our study revealed that neonatal survival rate is dramatically influenced by birth weight especially under 1000grams, GA especially below 30 weeks, neonatal anomalies, history of previous dead fetus, multiple pregnancy, non- cephalic presentation, and need for NICU admission, resuscitation and respiratory support with surfactant

## Introduction

Infants born before 37 weeks of gestation are at greater risk for morbidity and mortality compared with term newborns (1). Preterm births (PTB) have been increased approximately 30% since 1981 and comprised 12.5% of all births in year 2004 in United States (2). In industrialized countries, preterm delivery is responsible for 70% of neonatal mortalities and 75% of morbidities. In our country the most common cause of neonatal death is also prematurity, which accounts for 57.07% of neonatal death (3). It also contributes to significant long-term neuro-developmental problems, pulmonary dysfunction, and visual impairment (4, 5). Low birth weight (LBW) is also accounted for two-third of neonatal deaths (6). 

Although extremely low birth weight (ELBW) (≤1000 g) infants comprise less than 1% of all live births, they account for approximately half of the perinatal mortality rate (7). During the last 30 years, technological and pharmacological advances such as: antenatal steroid administration for lung maturation, initiation of assisted ventilation at delivery, surfactant therapy, new techniques of ventilation, and regionalization of perinatal care, have been resulted in a substantial increase in survival of preterm infants mainly at extremely low gestational ages (8). The most important contributing factors in mortality of preterm infants at different gestational ages (GA) are: neonatal sex and birth weight, GA at birth, vaginal delivery, antenatal corticosteroid administration; need for neonatal intensive care unit (NICU) admission and postnatal surfactant use (9-16). 

Neonatal outcome within different gestational ages and risk factors for neonatal mortality have been studied in many countries, but such comprehensive data are not available in Iran, and thus it is hard for healthcare professionals to provide reliable information for parents about outcomes of their preterm infants. Having knowledge about outcome, influences the professionals to take care of mothers and their infants. Our objective was to evaluate neonatal mortality rate of preterm infants, and to define its predictors and risk factors in Iranian population. This study is the second of this kind after the study by Fallahian *et al* in our population, with larger sample size and considering the risk factors of neonatal mortality (17). 

## Materials and methods

This historical cohort study was approved by the Institutional Research Board and the ethics committee of Iran University of Medical Sciences. This study had no financial support. It included all preterm (26-37 weeks) infants (n=1612) born alive in Shahid Akbar-abadi University Hospital, during one year period (April 2010-April 2011). This hospital is the largest maternity hospital in the capital city of Iran, Tehran, with a level III NICU. It covers more than 80% of all deliveries in the south and south west, with approximately 20,000 deliveries in a year. 

**Data Collection**

Data was collected using questionnaires from patient medical records. The questionnaire comprised of fetal-neonatal, maternal, and pregnancy data; antenatal medications; root of delivery; management in the neonatal period; postnatal outcomes (described below) and delivery to discharge duration. Questionnaire was completed for each preterm baby during the study period, (N=1800). Inclusion criteria was all live born preterm (before 37 weeks of gestation) babies delivered during the study period, with at least one examination in first 20 week (for accurate estimation of GA). Infants born from diabetic and hypertensive mothers, who had lethal congenital anomalies, who did not receive prenatal steroid (one or two doses of 12mg Betamethasone, 24 hours apart) at the time of admission and who received antenatal magnesium sulfate were excluded from the analysis. 

**Outcome Measures**

Primary outcome was delivery to discharge survival rate among preterm infants. Predictive variables for survival were: GA, presentation, order, delivery root, Apgar score, birth weight, neonatal sex, congenital non-fatal anomalies (including cardiovascular, urogenital, skeletal, gastrointestinal and central nervous system anomalies), need for NICU admission (defined as admitting in NICU for more than 12 hr), need for resuscitation (defined as need for either cardiac massage or tracheal intubation after birth), duration of NICU admission, postnatal surfactant (administered in cases of respiratory distress syndrome), time of neonatal death and time to discharge. 

Secondary outcomes were delivery to discharge survival for each predictor variable separately, the most important of them were: GA, neonatal birth weight, sex and Apgar score. GA was determined on the basis of the last menstrual period and sonography in the first half of pregnancy. According to current guidelines developed by the American Academy of Pediatrics, we did not initiate resuscitation for infants younger than 23 weeks or those whose birth weight was less than 400 g (18). 

Survival was defined as the proportion of live births surviving up to the time of discharge. Early and late neonatal mortality rates were defined as proportion of dead neonates in first 7 and 28 days respectively. LBW, VLBW and ELBW were defined as birth weight under 2500, 1500 and 100, respectively. Late PTB, early PTB and very early PTB were defined as delivery at 37-34, 34-30 and under 30 weeks of gestation.

**Statistical analysis**

Numerical variables were reported as mean±SD when normally distributed, otherwise as median and range. We used independent sample t-test and analysis of variance to compare the difference of numerical variable across demographic groups. The Chi-square was used for analysis of the qualitative variables. The Kaplan-Meier method was calculated to estimate mortality rate of preterm infants. The effect of GA, birth weight, neonatal sex, Apgar score on survival were studied by Kaplan-Meier survival estimators for each category using log rank test. P≤0.05 was considered to be statistically significant. All analyses were performed using SPSS for Windows version 16 (SPSS Inc., Chicago, IL, USA). 

## Results

Questionnaire was completed for 1800 preterm baby during the study period, among which 178 were excluded. Analysis was performed on the complete data of 1612 preterm neonates. Characteristics of the study group are summarized in table I. Mean GA at birth was 33.82±2.78 weeks and mean birth weight was 2262±617.85 grams. Among all, 147 neonates (9.1%) were died during hospital stay.

**Figure 1:**
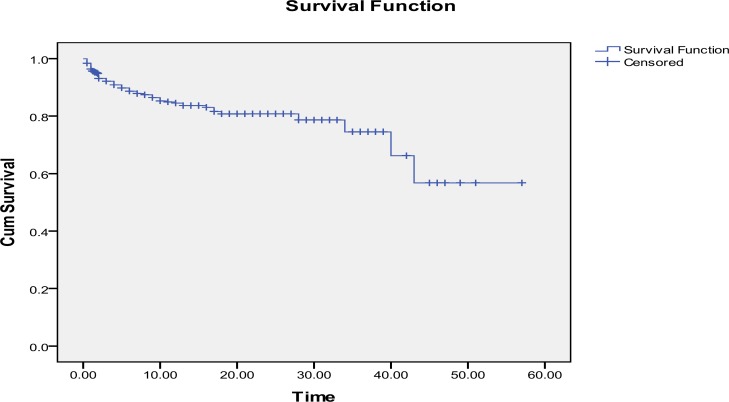
Total survival estimation of preterm neonates, limited to the largest survival time. Estimated mean of survival: 43.008±2.10 days (38.87-47.14). Cum survival: cumulative survival, Time: days spent after birth. Survival was estimated using Kaplan-Meier method.

Among dead neonates, 39% and 63.7% were died during the first 24 hours and the first 48 hours, respectively, 84.3% in early neonatal period (the first week), 12.24% in late neonatal period (7-28 days) and 3.4% died thereafter. Survival rate at first 24 hours, 48 hours, one week, 2 weeks and 4 weeks were 95%, 93%, 87%, 83%, and 79% respectively. Total cumulative survival of preterm neonates is demonstrated in figure1, Kaplan-Meier survival analysis. The mean survival time was estimated 43.008±2.10 days (38.87-47.14). 

All neonates with GA ≤25 weeks or birth weight ≤700 grams died before 48 hours, where, 11.11% (n=5 out of 45) of extremely LBW and 45.12% (n=74 out of 162) of very early PTBs were survived. The smallest surviving neonate was a 750 gr female with GA of 30 weeks and the youngest neonate was a 970 gr female with GA of 25 weeks plus 2 days. Survival was higher in late PTBs comparing early and very early PTBS (p=0.0001) and also higher in normal birth weights comparing LBW, VLBW and ELBW babies (p=0.0001). 

**Figure 2:**
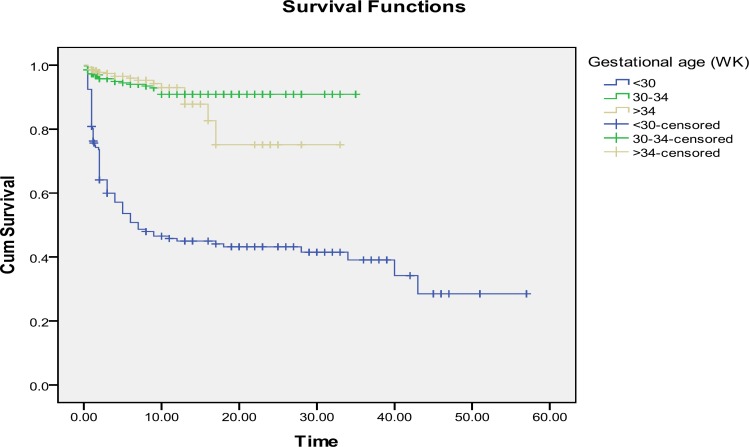
Survival estimation of preterm neonates according to gestational age at birth, limited to the largest survival time. Cum survival: cumulative survival, Time: days spent after birth. Survival was estimated using Kaplan-Meier method and Log-Rank test, p<0.0001.

**Figure 3:**
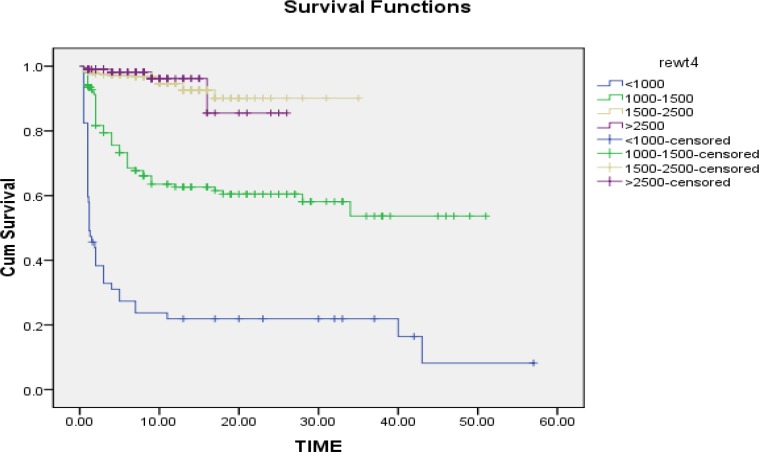
Survival estimation of preterm neonates according to birth weight, limited to the largest survival time. Cum survival: cumulative survival, Time: days spent after birth. Survival was estimated using Kaplan-Meier method and Log-Rank test, p<0.0001.

Survival for very early PTB and ELBW infants were dramatically low. Preterm neonates’ survival specified for GA and birth weight estimation using Kaplan-Meier method and Log-Rank test are shown in figures 2 and 3 respectively. Neonatal survival specified for GA and birth weight was calculated, as shown in table II. History of previous dead neonate (OR: 2.14, p=0.007), need to cardio-pulmonary resuscitation (CPR) (OR: 15.51, p=0.0001), need to NICU admission (OR: 1.93, p=0.0001), postnatal administration of surfactant (OR: 6.39, p=0.0001), presence of anomalies (OR: 5.20, p=0.0001), Apgar score <7 (OR: 30.84, p=0.0001), multiple pregnancy (OR: 1.53, p=0.0001), non-cephalic presentation (OR: 2.23, p=0.0001), early PTB (OR: 2.41, p=0.0001), very early PTB (OR: 10.7, p=0.0001), LBW (OR: 1.51, p=0.0001), VLBW (OR: 33.99, p=0.0001) and ELBW(OR: 43.5, p=0.0001), were risk factors for mortality in preterm neonates (Table II). 

We could not find a significant difference among male and female sex for mortality rate, however, neonates with ambiguous genitalia showed very high mortality rate (66.66%). Six neonates had ambiguous genitalia, 4 cases were among dead (2.7%) and 2 among alive neonates (0.1%). This difference was statistically significant (p=0.0001). There was also no difference in root of delivery and mother’s education among dead and alive neonates.

**Table I:** Demographic data of the study group

**Variable**									**Number (%)/ mean ± SD**
Maternal age (years)									26.90 ± 5.95 (14-63)
Primi gravida									824 (51.1)
Nulliparous									949 (58.9)
History of abortion									264 (16.4)
History of dead fetus									95 (5.9)
Mother’s education									
		Not educated							57 (3.5)
		Low educated (undergraduate)							811 (50.3)
		Educated (diploma and more)							744 (46.1)
Birth Order									
	Singleton								1274 (79)
	Twin								293 (18.2)
	Triple								45 (2.8)
Presentation at birth									
			Cephalic						1306 (81)
			Breech						249 (15.4)
			Transverse						20 (1.2)
			Other presentations						37 (2.3)
Mean Gestational age (weeks)									33.82 ± 2.78 (22.6-36.6)
Gestational age (weeks)									
				>34 (late preterm)					1007 (62.5)
				30-34(early preterm)					424(26.3)
				<30 (very early preterm)					181 (11.2)
Root of delivery									
					NVD				736 (45.7)
					C/S				876 (54.3)
Sex									
						Male			847 (52.5)
						Female			759 (47.1)
						Ambiguous			6 (0.4)
Apgar score <7									
							1-minute		123 (7.6)
							5-minute		86 (5.3)
CPR needed									202 (12.5)
NICU needed									670 (41.6)
Postnatal surfactant									110 (6.8)
Mean birth weight (gr)									2261 ± 617.85 (180-4100)
Birth weight (gr)									
								>2500 ( normal birth weight)	468 (29)
								<2500 (low birth weight)	908 (56.3)
								<1500 ( very low birth weight)	184 (11.4)
								<1000 (extremely low birth weight)	52 (3.2)
congenital malformation									70 (4.3)

NVD: normal vaginal delivery

C/S: cesarean section

CPR: cardio pulmonary resuscitation

NICU: neonatal intensive care unit.

**Table II:** Survival estimation specified for gestational age and birth weight using Kaplan-Meier method

			**Early Neonatal Survival**	**Neonatal Survival**
Total			87%	79%
Gestational age (weeks)				
	>34 (late preterm)		95%	75%
	30-34 (early preterm)		94%	90%
	<30 (very early preterm)		48%	41%
Birth weight (gram)				
		>2500 ( normal birth weight)	98%	85%
		1500-2500 (low birth weight)	96%	90%
		1000-1500 (very low birth weight)	68%	58%
		<1000 (extremely low birth weight)	24%	22%

Early Neonatal Survival: percent survived up to the end of first week

Neonatal Survival: percent survived up to the end of 28 days.

**Table III:** Comparison between alive and dead neonates

**Variable**		**Alive neonates** **[n=1465, n (%)]**	**Dead neonates** **[n=147, n (%)]**	**p-value**	**OR (CI 95%)**
History of previous dead child				0.007	2.14 (1.21-3.77
	Yes	79 (83.2)	16 (16.8)		
	No	1386 (91.4)	131 (8.6)		
CPR needed				0.0001	15.51(12.37-19.45)
	Yes	79 (39.1)	123 (60.9)		
	No	1386 (98.3)	24 ( 1.7)		
NICU needed				0.0001	1.93 (1.72-2.17)
	Yes	561 (83.7)	109 (16.3)		
	No	904 (96)	38 (4)		
Postnatal surfactant				0.0001	6.39 (4.53-9.01)
	Yes	67 (60.9)	43 (39.1)		
	No	1398 (93.1)	104 (6.9)		
Neonatal anomaly				0.0001	5.20 (3.27-8.26)
	Yes	46 (65.7)	24 (34.3)		
	No	1491 ( 92)	123 (8)		
5- min Apgar <7				0.0001	30.84 (19.43-48.95)
	Yes	21 (24.4)	65 (75.6)		
	No	1444 (94.6)	82 (5.4)		
Multiple pregnancy				0.003	1.53 (1.17-1.99)
	Yes	293 (86.7)	45 (13.3)		
	No	1172 (92)	102 (8)		
non-cephalic presentation				0.0001	2.23 (1.76-2.82)
	Yes	250 (81.7)	56 (18.3)		
	No	1215 (93)	91 (7)		
Early preterm birth (GA<34w)				0.0001	2.41 (2.16-2.70)
	Yes	459 (79.8)	116 (20.2)		
	No	928 (97)	29 (3)		
Very early preterm birth (GA<30w)				0.0001	10.7 (8.6-13.7)
	Yes	81 (47.1)	91 (52.9)		
	No	1306 (96)	54 (4)		
Low birth weight (<2500 g)				0.0001	1.51 (1.43-1.61)
	Yes	864 (87.3)	126 (12.7)		
	No	530 (98.5)	8 (1.5)		
Very low birth weight (<1500 g)				0.0001	33.99 (22.01-52.5)
	Yes	111 (52.6)	100 (47.4)		
	No	1283 (97.4)	34 (2.6)		
Extremely low birth weight (<1000 g)				0.0001	43.5 (23-81.9)
	Yes	11 (19.3)	46 (80.7)		
	No	1383 (94)	88 (6)		

CPR: cardio pulmonary resuscitation

NICU: neonatal intensive care unit

GA: gestational age

OR (CI 95%): Odds ratio with 95% confidence interval.

p-values were obtained from χ-square test.

## Discussion

The present study revealed that LBW, VLBW, ELBW, early PTB, very early PTB, ambiguous gender, presence of anomalies, history of previous dead fetus, multiple pregnancy, non-cephalic presentation, need for NICU admission, need for resuscitation and respiratory support with surfactant, were associated with neonatal mortality in preterm births. It suggests that neonatal short term survival was dramatically influenced by birth weight especially ELBWs and GA especially below 30 weeks. It is generally accepted that births before 26 weeks, especially those weighting less than 750 gr, are at the current threshold of viability and that these preterm infants pose a variety of complex medical, social, and ethical considerations (18). The birth weight and gestational age threshold of viability in our study were 750 gr and 25 weeks plus 2 days. 

The smallest surviving infant was a 750 gram female with gestational age of 30 weeks and the youngest infants was a 970 gram female with GA of 25weeks plus 2 days. The same viability threshold was previously reported by Fallahian *et al*. in Iran, in a study with a small sample size, which showed that the expected survival rate of neonates was greatly influenced by a gestational age of higher than 32 weeks and a birth weight of more than 1250 grams (17). Consistent with our findings, this study revealed that an increase in the age and weight of preterm neonates leads to a rapid decline in mortality rate. 

The overall mortality rate of our study population was 9.1%. In a recent study in Qatar the mortality rate of 28-32 weeks infants was 65.3% which is much higher than our results (9). In another study on mortality rate for late preterm babies, the highest mortality rate was reported on 34 weeks neonates which were 13.5% and 12.9% for male and females, respectively (16). Lower mortality rate in our study could be for the lack of long term outcome evaluation of survived babies as we could not follow neonates after discharge. In a study conducted in Norway, high survival rate (59%) was reported for preterm babies born between 22-27 weeks (12). The survival rates for these infants were as follows:0% for <23 weeks, 16% for 23 weeks, 44% for 24 weeks, 66% for 25 weeks, 72% for 26 weeks, 82% for 27 weeks, and 69% for >27 weeks. However, in our study all infants with GA ≤25 weeks or birth weight ≤700 grams died before 48 hours and only 11.11% of ELBW (<1000 gr) and 45.12% of very early PTBs were survived. In a study on VLBWs (<1500 gr) in Switzerland the overall mortality was reported 13%, which is much less than ours (14). Short term mortality rate of preterm infants, particularly very early PTBs, in our country is much higher than developed countries. 

One study in our country showed that two-third of all neonatal deaths is occured in LBWs. Neonatal mortality rate in LBW, VLBW and ELBW neonates were 23, 62.5 and 117 times more than that of normal weight newborns, respectively (6). In other study in our county, survival rates for the groups of LBW and VLBW neonates were 98.4% and 66.6%, respectively. They showed that birth weight has the most effect on the survival rate among variables studied in their research (19). In another retrospective study in our county on newborns with a birth weight of ≤1500 g and a gestational age of ≤30 weeks who had been hospitalized during a 15-month period in NICUs, the mortality rate was reported 64.4% and the most common morbidity was respiratory distress syndrome (76%) (20).

In spite of modern neonatal management, male infants still seem to have higher mortality and poorer long-term neurologic outcome. We could not find a significant difference between male and female sex with regard to short term mortality rate; however neonates with ambiguous genitalia showed very high mortality rate. Gender differences for mortality and long-term neurologic outcome appear to lose its significance at 27 weeks gestation, and the results obtained in our study may be due to the higher gestational age of our study population (21). As we showed, mortality rate increases with decreasing GA. Late PTB has previously shown to be associated to increased risk of hospital death compared with delivery at higher gestational ages. Early and very early preterm infants have even more mortality rates (22). 

The risks of PTB with abnormal birth weight categories at both extreme (intrauterine growth restriction and large for gestational age) are 2-3 fold greater than the risk among appropriate for gestational age infants (10, 16, 23). Similarly, the results of present study showed a significant high mortality rate in LBW neonates, which was increased with decreasing weight and reached its nadir in ELBW infants. Cesarean section does seem to provide advantages for survival in most immature infants delivered at 22-25 weeks of gestation, independent of maternal risk factors for cesarean section (23). In our study, evaluation of the effect of root of delivery on survival of preterm infants between 22-31 weeks, showed 52.8% and 47.3% mortality rate for cesarean and vaginal deliveries, respectively. 

In a recent study on neonatal outcomes in early PTBs by delivery root, there was no difference in neonatal mortality for vertex presentation; however, neonatal mortality was increased for breech presentation in vaginal delivery (24). It also has shown that, vaginal delivery of 1,000-1,500 gr babies presenting as breech is associated with the increased neonatal mortality compared with cesarean delivery (25). However, our study showed no difference in the survival of neonates delivered by either vaginal or abdominal delivery. This also could be as a result of higher gestational age and higher proportion of cephalic presentation in our study population and different categorization in comparison with previous studies. 

We showed that the survival of neonates with cephalic presentation, either delivered vaginal or abdominal, were better than other presentations. It is clearly due to the more prevalence of non-cephalic presentation in smaller age and weight neonates, and more prevalence of anomalies in non-cephalic infants. In our study singletons had better survival compared to multiple pregnancies, while multiple births showed to have no detectable effect on the adjusted composite neonatal outcome of mortality and morbidities (13). Our study showed that history of at least on previous fetal death would increase the risk of fetal mortality in subsequent pregnancies, which could be due to inherited or genetic fetal reasons. 

The significant reduction in neonatal mortality during recent years, results from technological and pharmacological advances over the last 30 years. Considering the limitation of available resources, the important question is: where and how should we invest in neonatal care. Having knowledge about outcome of preterm infants and the risk factor of their mortality and morbidities influence the way professionals take care of mothers and their infants, and help to use the resources properly. The strength of this study was its large size and its novelty in our population. However, our data lacks the evaluation of neonata morbidities and long term outcome of survived babies as we could not follow neonates after discharge. The results also may not be generalizable to other populations. Preterm newborns from other populations may have different survival rates and mortality risk factors.

## Conflict of interest

The authors report no conflicts of interest. The authors alone are responsible for the content and writing of the paper.
